# Bakuchiol Suppresses Inflammatory Responses Via the Downregulation of the p38 MAPK/ERK Signaling Pathway

**DOI:** 10.3390/ijms20143574

**Published:** 2019-07-22

**Authors:** Hye-Sun Lim, Yu Jin Kim, Bu-Yeo Kim, Soo-Jin Jeong

**Affiliations:** 1 Herbal Medicine Resources Research Center, Korea Institute of Oriental Medicine, Naju-si, Jeollanam-do 58245, Korea; 2 Clinical Medicine Division, Korea Institute of Oriental Medicine, Daejeon 34054, Korea; 3 College of Pharmacy, Chungnam National University, Daejeon 34134, Korea

**Keywords:** bakuchiol, inflammatory response, MAPK phosphorylation, microglia, neuroinflammation

## Abstract

The purpose of the present study was to evaluate the effects of bakuchiol on the inflammatory response and to identify the molecular mechanism of the inflammatory effects in a lipopolysaccharide (LPS)-stimulated BV-2 mouse microglial cell line and mice model. The production of prostaglandin E_2_ (PGE_2_), tumor necrosis factor-α (TNF-α), and interleukin-6 (IL-6) was measured by enzyme-linked immunosorbent assay. The mRNA expression of inducible nitric oxide synthase (iNOS), cyclooxygenase-2 (COX-2), TNF-α, and IL-6 was measured using reverse transcription–polymerase chain reaction analysis. Mitogen-activated protein kinase (MAPK) phosphorylation was determined by western blot analysis. In vitro experiments, bakuchiol significantly suppressed the production of PGE_2_ and IL-6 in LPS-stimulated BV-2 cells, without causing cytotoxicity. In parallel, bakuchiol significantly inhibited the LPS-stimulated expression of iNOS, COX-2, and IL-6 in BV-2 cells. However, bakuchiol had no effect on the LPS-stimulated production and mRNA expression of TNF-α or on LPS-stimulated c-Jun NH2-terminal kinase phosphorylation. In contrast, p38 MAPK and extracellular signal-regulated kinase (ERK) phosphorylation were inhibited by bakuchiol. In vivo experiments, Bakuchiol reduced microglial activation in the hippocampus and cortex tissue of LPS-injected mice. Bakuchiol significantly suppressed LPS-injected production of TNF-α and IL-6 in serum. These results indicate that the anti-neuroinflammatory effects of bakuchiol in activated microglia are mainly regulated by the inhibition of the p38 MAPK and ERK pathways. We suggest that bakuchiol may be beneficial for various neuroinflammatory diseases.

## 1. Introduction

Inflammation plays a role in the pathology of neurodegenerative diseases and is dependent on the production of various inflammatory mediators by resident macrophages, such as microglia [1,2]. Microglia are the resident immune cells in the central nervous system and are activated by stimuli such as lipopolysaccharide (LPS), interferon-γ, or β-amyloid [3,4]. Activated microglia produce inflammatory mediators, including nitric oxide (NO) and prostaglandin E_2_ (PGE_2_), and proinflammatory cytokines, such as the tumor necrosis factor-α (TNF-α), interleukin (IL)-1β, and IL-6 [5]. Moreover, the activation of microglia is mediated by cellular kinases, such as mitogen-activated protein kinases (MAPKs). MAPKs, such as p38 MAPK, extracellular signal-regulated kinase (ERK), and c-Jun N-terminal kinase (JNK), are involved in the transcriptional regulation of inflammatory factors. Overproduction of these inflammatory molecules in microglia causes neurodegenerative diseases such as Alzheimer’s disease, Parkinson’s disease, and trauma [5,6]. Therefore, the regulation of microglial activation is an important therapeutic approach for the treatment of inflammation-related neurodegenerative diseases.

**Figure 1:**
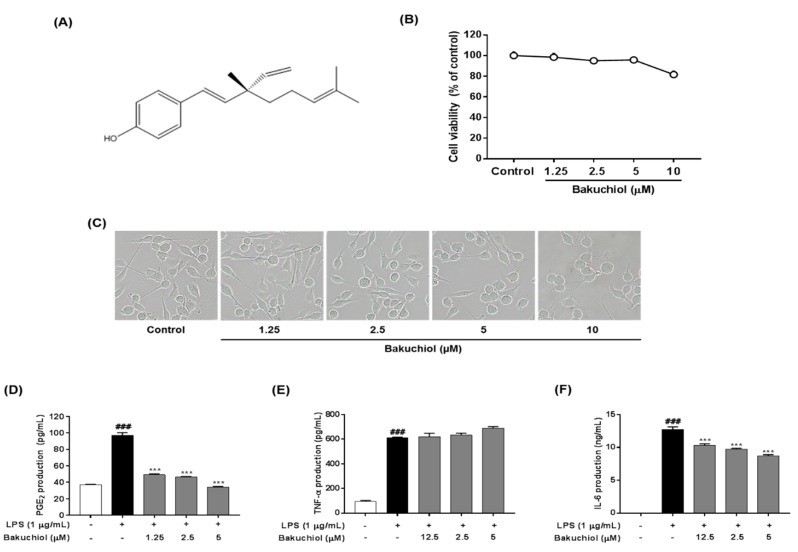
Cytotoxicity and effects of bakuchiol on lipopolysaccharide (LPS)-stimulated prostaglandin E_2_ (PGE_2_), tumor necrosis factor-α (TNF-α), and interleukin-6 (IL-6) production of bakuchiol in BV-2 microglia. Chemical structure of bakuchiol (**A**). Cells were seeded on 96-well plates and treated with various concentrations (0, 1.25, 2.5, 5, or 10 μM) of bakuchiol for 24 h (**B**). Cell viability was assessed using the CCK-8 assay. Morphological assessment after bakuchiol treatment. Images are shown from cells treatment 24 h to 0, 1.25, 2.5, 5, or 10 μM bakuchiol (**C**). Photomicrographs were taken under a standard light microscope at 20× magnification. The production of PGE_2_ (**D**), TNF-α (**E**), and IL-6 (**F**) was measured in the culture medium and assessed using enzyme-linked immunosorbent assay (ELISA). The results are expressed as the mean ± SEM of three independent experiments. ^###^ *P* < 0.001 vs. untreated control cells and *** *P* < 0.001 vs. LPS-treated cells.

Bakuchiol (Figure 1A) is the main chemical constituent of the seeds of *Psoralea corylifolia, * which has been used in traditional Oriental medicine to treat lung adenocarcinoma [7], bone loss [8], and oxidative stress [9]. Many researchers have reported that bakuchiol possesses several pharmacological effects, such as anti-tumor [7], anti-microbial [10], anti-aging [11], and anti-inflammatory effects [12]. Although Choi et al. reported the anti-inflammatory action of bakuchiol, this effect was demonstrated using macrophages, and not glia in brain, without investigation of its regulatory molecular mechanisms. We have recently reported quantitative analyses of the seven standard components of *P. corylifolia*, psoralen, angelicin, neobavaisoflavone, psoralidin, isobavachalcone, bavachinin, and bakuchiol, and examined whether the seven components influenced the production of nitric oxide (NO) in LPS-stimulated BV-2 microglia and had a neuroprotective effect in HT22 hippocampal cells [13]. Our findings suggest that the most significant effects of bakuchiol compared with other components are its anti-neuroinflammatory and neuroprotective actions. Based on our previous study, we expanded our investigation of the inhibitory effects of bakuchiol on LPS-stimulated production and expression of inflammatory mediators and cytokines in the BV-2 microglial cell line. We also investigated the molecular mechanisms that underlie the bakuchiol-mediated regulation of inflammation via the targeting of MAPK signaling in BV-2 cells. In addition, we determined the anti-inflammatory effect of bakuchiol in an LPS-injected neuroinflammation mouse model.

## 2. Results

### 2.1. Effect of Bakuchiol on the Viability of BV-2 Microglia

The effect of bakuchiol on cell viability was measured using various concentrations of the compound, ranging from 1.25 to 10 μM, for 24 h. Nontoxic concentrations (>90% of cell viability compared with the control) were determined for the subsequent biological assays. As shown in Figure 1B,C, cytotoxicity of bakuchiol was observed at 10 μM (81.62% ± 3.37%), whereas no toxicity was detected at a concentration ≤5 μM.

### 2.2. Bakuchiol Inhibits the Production of PGE_2_ and IL-6, But not TNF-α, in LPS-induced BV-2 Microglia

To determine the effects of bakuchiol on the production of PGE_2_ and the inflammatory cytokines IL-6 and TNF-α after LPS stimulation, BV-2 cells were pretreated with various concentrations of bakuchiol (0, 1.25, 2.5, or 5 μM) for 2 h and then stimulated with LPS (1 μg/mL) for an additional 22 h. As shown in Figure 1D, LPS treatment significantly increased PGE_2_ production compared with untreated cells. In contrast, bakuchiol significantly inhibited the PGE_2_ production induced by LPS stimulation. The production of TNF-α and IL-6 was significantly increased in LPS-stimulated BV-2 cells compared with untreated cells. Bakuchiol significantly decreased the production of IL-6 in a dose-dependent manner, but LPS-stimulated TNF-α production was not affected in BV-2 cells (Figure 1E,F).

### 2.3. Bakuchiol Inhibits mRNA Expression of iNOS, COX2, and IL-6, But not TNF-α, in LPS-induced BV-2 Microglia

**Figure 2:**
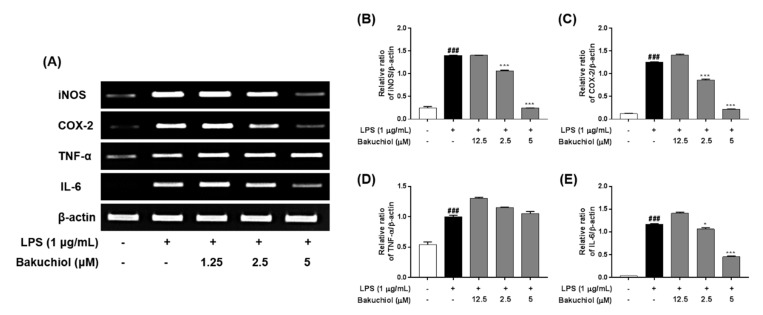
Effects of bakuchiol on the LPS-stimulated mRNA expression of inflammatory molecules in BV-2 microglia. Total RNA was prepared for the RT–PCR analysis of iNOS, COX-2, TNF-α, and IL-6 mRNA expression (**A**). Bar graphs represent the relative mRNA expression of iNOS (**B**), COX-2 (**C**), TNF-α (**D**), and IL-6 (**E**) adjusted to β-actin expression. The results are expressed as the mean ± SEM of three independent experiments. ^###^*P* < 0.001 vs untreated control cells and * *P* < 0.05 or *** *P* < 0.001 vs LPS-treated cells.

To investigate the inhibitory effects of bakuchiol on inflammation-related factors, the mRNA expression of inflammatory molecules was analyzed by RT–PCR. BV-2 cells were pretreated with bakuchiol for 1 h and then stimulated with LPS (1 μg/mL) for 5 h. As shown in Figure 2, LPS treatment induced a dramatic increase in iNOS, COX-2, TNF-α, and IL-6 compared with untreated cells. Consistent with the results depicted in Figure 1, bakuchiol inhibited iNOS, COX-2, and IL-6 mRNA expression occurred in a dose-dependent manner in LPS-stimulated BV-2 cells. However, TNF-α mRNA expression was not affected by the drug, which was similar to that observed for TNF-α production.

### 2.4. Bakuchiol Inhibits the Phosphorylation of p38 MAPK and ERK, but not of JNK, in LPS-induced BV-2 Microglia

**Figure 3:**
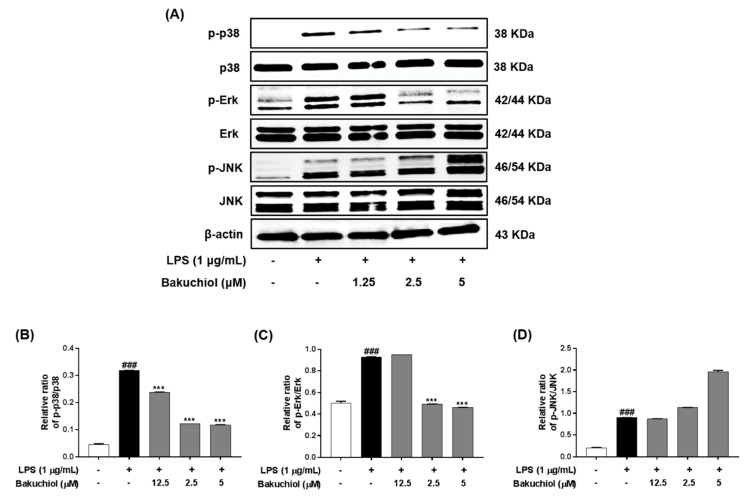
Effects of bakuchiol on mitogen-activated protein kinase (MAPK) phosphorylation in LPS-stimulated BV-2 microglia. The cellular proteins were used for the detection of phosphorylated or total forms of p38 MAPK, extracellular signal-regulated kinase (ERK), and c-Jun N-terminal kinase (JNK) by western blotting (**A**). Bar graphs represent the relative phosphorylation of the p38 MAPK (**B**), ERK (**C**), and JNK (**D**) proteins adjusted to the β-actin protein level. The results are expressed as the mean ± SEM of three independent experiments. ^###^ *P* < 0.001 vs untreated control cells and *** *P* < 0.001 vs LPS-treated cells.

**Figure 4:**
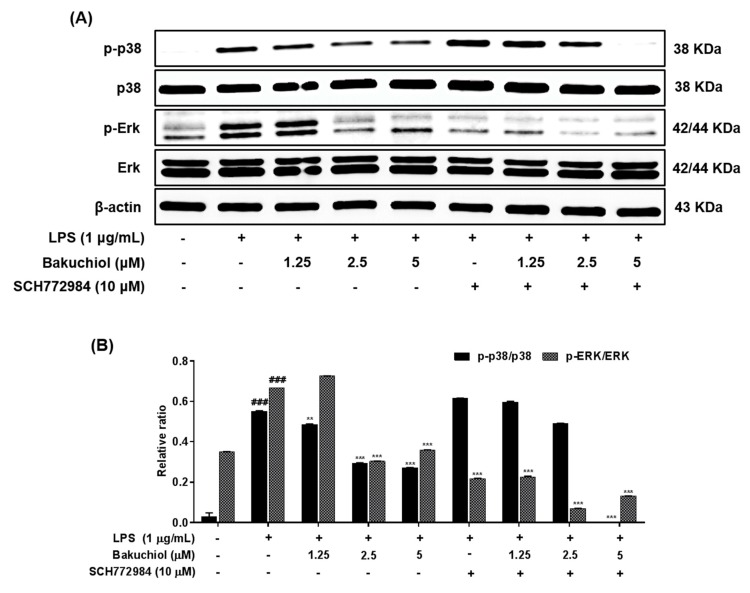
Effects of bakuchiol on p38 MAPK and ERK phosphorylation in LPS-stimulated BV-2 microglia. The cellular proteins were used for the detection of phosphorylated or total forms of p38 MAPK and ERK by western blotting (**A**). Bar graphs represent the relative phosphorylation of the p38 MAPK and ERK (**B**) proteins adjusted to the β-actin protein level. The results are expressed as the mean ± SEM of three independent experiments. ^###^ *P* < 0.001 vs untreated control cells and ^**^P < 0.01 or *** *P* < 0.001 vs LPS-treated cells.

To clarify the molecular mechanism underlying the anti-inflammatory effects of bakuchiol, we analyzed the phosphorylation of p38 MAPK, ERK, and JNK by western blotting. As shown in Figure 3A,B, LPS treatment remarkably elevated the phosphorylation of p38 MAPK, ERK, and JNK. In addition, the phosphorylation of p38 MAPK and ERK was remarkably attenuated by bakuchiol, whereas the phosphorylation of JNK was not affected by the drug. These results indicate that bakuchiol blocked the p38 MAPK and ERK pathways to exert an anti-inflammatory effect in LPS-stimulated BV-2 cells. To determine if activation of either MAPK contributed to the anti-neuroinflammatory action of bakuchiol, we used SCH772984, a selective inhibitor of ERK. As shown in Figure 4A,B, SCH772984 inhibited bakuchiol-induced p38 MAPK and ERK pathways, implying that they play a role in LPS-induced inflammation.

### 2.5. Effect on Bakuchiol on Microglial Activation and the Production of TNF-α and IL-6 in LPS-induced Mice

**Figure 5:**
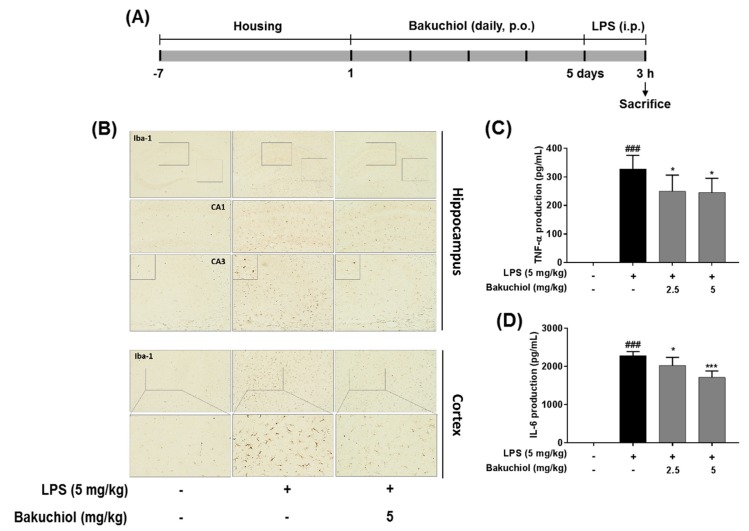
Effects of bakuchiol on neuroinflammation in LPS-induced mice. Mice were assigned to four groups (*n* = 7/group) and administered vehicle (saline) or bakuchiol at doses of 2.5 or 5 mg/kg orally for 5 days after adaptation for 1 week. On day 5, LPS (5 mg/kg) were administered 3 h before the sacrificed (**A**). Immunoreactive cells of anti-Iba1 antibody was investigated in the brain hippocampus and cortex by immunohistochemistry analysis (**B**). Production of TNF-α (**C**) and IL-6 (**D**) in the serum were measured using ELISA. The results are expressed as the mean ± SEM of three independent experiments. ^###^ *P* < 0.001 vs untreated control cells and * *P* < 0.05 or *** *P* < 0.001 vs LPS-treated cells.

To further examine the inhibitory effects of TEC on inflammatory responses identified in vitro, LPS treatment in a murine model of inflammation was used to study the suppressive effects of bakuchiol on neuroinflammation in vivo. Microglial cells were immunostained with the Iba-1. LPS-treated mice demonstrated increased expression of Iba-1 compared to vehicle-treated controls; however, expression of Iba-1 were decreased in bakuchiol-treated mice (Figure 5B). In addition, as shown in Figure 5C,D, production of TNF-α and IL-6 in the serum were significantly increased in LPS-treated mice compared to vehicle-treated control mice.

## 3. Discussion

Brain inflammation has been implicated as a critical mechanism in the progression of neurodegeneration and is characterized by the activation of microglia accompanied by the production of inflammation-related factors [14]. Microglia are resident macrophages in the brain that contribute to neuroinflammation. Activated microglia secrete inflammatory mediators and cytokines, and these lead to neuronal damage, which is the basis of a large variety of brain pathologies [15]. Therefore, the development of agents that have the ability to inhibit inflammatory factor production may be a promising therapeutic approach for the treatment of neurodegenerative diseases.

**Figure 6:**
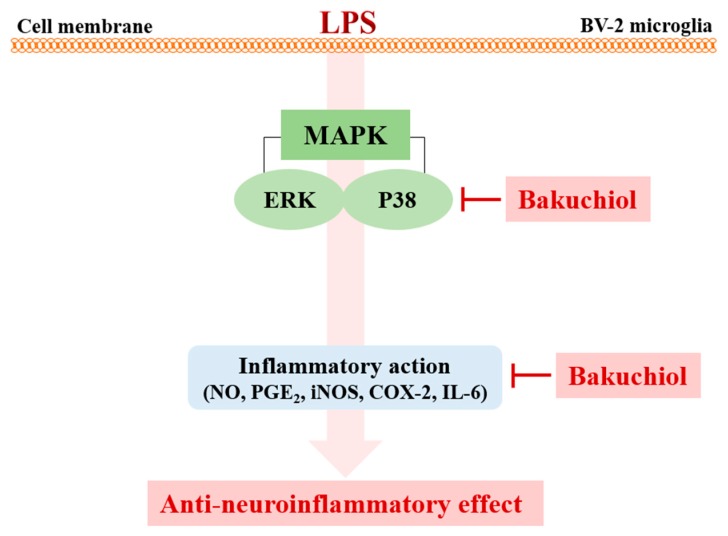
Schematic model of the anti-neuroinflammatory modulation afforded by bakuchiol in activated microglia. Bakuchiol inhibits LPS-stimulated neuroinflammation through the p38 MAPK and ERK signaling pathways.

Recently, natural compounds isolated from medicinal plants have drawn attention regarding the treatment of various diseases. Plant products are generally considered to be less toxic and have fewer side effects compared with chemically synthesized drugs [16]. Na Liu et al. reported that Schisandrin B isolated from *Schisandra chinensis* exhibited anti-inflammatory effects in BV2 microglia via the activation of PPAR-γ [17]. In addition, Jeong YH et al. reported that the natural compound lonchocarpine inhibits microglial activation by modulating various proinflammatory molecules and signaling pathways in TLR3 or TLR4 agonist-induced neuroinflammation models [18]. Therefore, plant-derived materials have received increased attention as biochemically active agents in the therapies of many diseases, including neuroinflammation [19]. In this study, we investigated whether bakuchiol isolated from the seeds of *P. corylifolia* has anti-inflammatory activity in brain cells via the modulation of the p38 MAPK/ERK pathways and microglia inactivation in mice with lipopolysaccharide (Figure 6). Microglia, which are key innate immune cells of the brain, exhibit increased expression of iNOS and COX-2. These are important enzymes that are responsible for the production of NO and PGE_2_ during the inflammatory process induced by LPS stimulation. Inflammatory mediators are responsible for the harmful effects observed in brain diseases such as Alzheimer’s disease and Parkinson’s diseases and for neuronal cell death [20]. Therefore, targeting the inflammatory mediators NO and PGE_2_ has been considered a useful therapeutic strategy for the treatment of neuroinflammatory diseases [21]. Our previous report demonstrated that bakuchiol inhibited the production of NO compared with other chemical constituents of *P. corylifolia* [13]. Based on these results, in the present study, we further investigated whether bakuchiol inhibits LPS-mediated neuroinflammatory responses and its regulatory mechanisms using BV-2 microglia. Bakuchiol significantly inhibited the production of PGE_2_ in LPS-stimulated BV-2 cells (Figure 1C). Moreover, we provide evidence that bakuchiol-mediated inhibition of NO and PGE_2_ production was the consequence of the decrease in the mRNA levels of iNOS and COX-2 in LPS-stimulated BV-2 cells (Figure 2A–C). Thus, the present findings suggest that bakuchiol has protective effects against neuroinflammation.

Proinflammatory cytokines, including TNF-α and IL-6, are produced after microglial activation and their abnormal production or functions in the brain are characteristic of various neurological diseases [22]. TNF-α and other proinflammatory mediators and neurotoxic substances are generally produced by activated microglia [23]. IL-6 is a multifunctional cytokine that plays an important role in host defense, with major regulatory effects on the inflammatory response [24]. In this study, we investigated whether bakuchiol inhibits the LPS-stimulated production of these cytokines. Our results indicated that bakuchiol inhibited LPS-stimulated IL-6 production in BV-2 cells, but did not affect TNF-α production (Figure 1D,E). We also found that the inhibitory effects of bakuchiol on LPS-stimulated cytokine production were correlated with its ability to suppress their expression at the gene level. The mRNA expression of IL-6 was markedly reduced after bakuchiol administration (Figure 2A,E), whereas the expression of TNF-α was not affected in LPS-activated cells (Figure 2A,D).

Various intracellular signaling pathways are involved in inflammatory regulation in the brain. Several inflammatory stimuli, such as LPS, interferon-γ, or β-amyloid, commonly activate the nuclear factor-kappa B, MAPK, and phosphatidylinositol-3-kinase pathways in microglia. Among them, MAPK pathways are the most critical regulators of proinflammatory cytokines and play a role in initiating and sustaining inflammatory reactions [25,26]. Many previous studies have shown that the activation of MAPKs has a significant effect on the regulation of COX-2, iNOS, and proinflammatory cytokines in microglia [27]. Hence, it is possible that the anti-neuroinflammation mechanisms of bakuchiol are associated with the inhibition of the MAPK signaling pathway in activated microglia. In the present study, we found that bakuchiol inhibited the activation of p38 MAPK and ERK, but not of JNK, in response to LPS in BV-2 cells, suggesting that p38 MAPK and ERK are important molecular targets of bakuchiol (Figure 3). Here, we showed that cotreatment with bakuchiol and the ERK inhibitor SCH772984 further suppressed the activation of p38 MAPK and ERK compared to bakuchiol alone in LPS-stimulated BV-2 cells (Figure 4). These results also highlight the possibility that the bakuchiol-mediated attenuation of the inflammatory mediators such as iNOS, COX-2, and IL-6 is correlated with the downregulation of the MAPK signaling pathways in BV-2 microglia, as evidenced by the suppression of the phosphorylation of p38 MAPK and ERK by bakuchiol.

We examined the inhibitory effects of bakuchiol on neuroinflammation using mouse model by LPS administration. Iba-1 plays important role in the regulation of several immunological functions of microglia and serves as a unique marker for detecting microglial activation [28]. Bakuchiol diminished the morphological changes indicative of activated microglia form that increased cell size, irregular shape, and intensified in the hippocampus and cortex through Iba-1 immunostaining (Figure 5B). Bakuchiol also significantly inhibited the production of TNF-α and IL-6 in serum compared to LPS group in dose-dependent (Figure 5C,D). These effects were consistent with those observed in our in vitro experiments.

Overall, our previous and present results imply that bakuchiol modulates the suppression of IL-6, iNOS, and COX-2 expression, concomitant with the inhibition of IL-6, NO, and PGE_2_ production. Bakuchiol has been shown to suppress microglial inflammatory responses against the LPS-induced activation of the p38 MAPK and ERK signaling pathways (Figure 5). These results suggest that bakuchiol may represent a therapeutic intervention for inflammatory-mediated neurodegenerative diseases. Future studies will be necessary to further determine the key molecular mechanisms of bakuchiol for treating neuroinflammatory diseases using in vitro or in vivo models.

## 4. Materials and Methods

### 4.1. Cell Culture, Animals, and Drug Administration

The mouse BV-2 microglial cell line was maintained in Dulbecco’s Modified Eagle’s Medium (Hyclone/Thermo, Rockford, IL, USA) supplemented with 10% fetal bovine serum (Hyclone/Thermo) and penicillin/streptomycin at 37 °C under 5% CO_2_. Male ICR mice (8 weeks old) were purchased from Dae Han Biolink (Eumseong, Korea). All animal experiments were approved by the Institutional Animal Care and Use Committee of the Korea Institute of Oriental Medicine (Approval No. 17-038; March 10, 2017) and carried out in accordance with the dictates of the National Animal Welfare Law of Korea. Mice were divided into 4 groups (*n* = 7/group): (1) saline + vehicle control group; (2) saline + LPS group; (3) bakuchiol (2.5 mg/kg) + LPS group; (4) bakuchiol (5 mg/kg) + LPS group. Bakuchiol was administered per os (p.o.) once per day for 5 days and LPS was injected intraperitoneally (5 mg/kg) 3 h after the final bakuchiol administration (Figure 5A).

### 4.2. Cytotoxicity Assay

Cytotoxicity was performed using a Cell Counting Kit-8 (CCK-8; Dojindo, Kumamoto, Japan) according to the manufacturer’s protocol. In brief, BV-2 cells were seeded on 96-well microplates at a density of 3 × 10^4^ cells/well and treated with bakuchiol (0, 1.25, 2.5, 5, or 10 μM; Shanghai Sunny Biotech Co., Shanghai, China) for 24 h. Then, 10 μL CCK-8 solution were added to each well for an incubated for 4 h. Absorbance was determined at 450 nm using a Benchmark Plus microplate reader (Bio-Rad Laboratories, Hercules, CA, USA).

### 4.3. Enzyme-linked Immunosorbent Assay for PGE_2_, TNF-α, and IL-6

BV-2 cells were pretreated with bakuchiol for 2 h and then stimulated with LPS (1 μg/mL) for 22 h, and then, supernatant of the cell culture was collected and centrifuged. Samples were applied to each well for enzyme-linked immunosorbent assay (ELISA). The PGE_2_ were detected using an ELISA kit from Cayman Chemical Company (Ann Arbor, MI, USA), TNF-α and IL-6 were measured by ELISA kit from R&D Systems (Minneapolis, MN, USA) according to the manufacturer’s protocols.

### 4.4. RNA Extraction and Reverse Transcription–Polymerase Chain Reaction Analysis

Total RNA was extracted from BV2 cells using the TRIzol reagent (Invitrogen Co., Carlsbad, CA, USA). Total RNA (1 μg) were reverse transcribed into cDNA using an iScript cDNA synthesis kit (Bio-Rad, Hercules, CA, USA) according to the manufacturer’s protocols. As described previously [29], polymerase chain reaction (PCR) was performed using cDNA as a template for amplification conditions. The primer list is provided in Table 1. The PCR products were separated on a 1.5% agarose gel electrophoresis using Azure C150 Gel Imaging Workstation (Azure Biosystems, Dublin, CA, USA). The relative expression levels of iNOS, COX-2, TNF-α, and IL-6 were adjusted based on the expression of β-actin, which was used as a control.

### 4.5. Western Blot Analysis

Western blot analysis procedures were carried out as our previously described [29]. The polyvinylidene fluoride (PVDF) membranes were incubated overnight with primary antibodies: Against phosphorylated or total forms of p38 MAPK, ERK, JNK (rabbit polyclonal antibodies, 1:1000 dilution; Cell Signaling Technology, Danvers, MA, USA) and against β-actin (rabbit polyclonal antibodies, 1:1000 dilution; Cell Signaling Technology). PVDF membranes were washed three times with tris-buffered salient containing 0.5% tween 20 and incubated with a 1:3000 dilution of a horseradish peroxidase-conjugated secondary antibody (Jackson ImmunoResearch, West Grove, PA, USA) for 1 h at room temperature. Images of developed antibody specific bands were captured using an LAS 4000 mini luminescent image analyzer (GE Healthcare Bio-Sciences, Piscataway, NJ, USA).

### 4.6. Immunohistochemistry

Brains were sectioned via a freezing microtome (Leica Instruments GmbH, Nussloch, Germany). Brain tissue fixation on cover slips and further steps were carried out as our previously described [29]. Detection of the Iba-1 was performed using a microscope (Olympus Microscope System CKX53; Olympus, Tokyo, Japan).

### 4.7. Statistical Analysis

Graph Pad software version 7 (Graph Pad Software, La Jolla, CA, USA) were used to carry out statistical analyses. Data are expressed as the mean ± SEM of three separate experiments and were analyzed using one-way analysis of variance followed by Dunnett’s multiple comparisons test. *P* < 0.05 were considered to indicate statistical significance.

## Figures and Tables

**Table 1:** Primer sequences for reverse transcription-polymerase chain reaction.

Gene	Primer Sequences	
*iNOS*	Forward	3′-CCTCCTCCACCCTAGCAAGT-5′
	Reverse	3′-CACCCAAAGTGCTTCAGTCA-5′
*COX-2*	Forward	3′-AAGACTTGCCAGGCTGAACT-5′
	Reverse	3′-CTTCTGCAGTCCAGGTTCAA-5′
*TNF-α*	Forward	3′-TGGGTAGAGAATGGATGAAC-5′
	Reverse	3′-GCCGATTTGGTATCTCATAC-5′
*IL-6*	Forward	3′-AAGAGACTTCCATCCAGTTG-5′
	Reverse	3′-TCCAGGTAGCTATGGTACTC-5′
*β-actin*	Forward	3′-TGTGATGGTGGGAATGGGTCAG-5′
	Reverse	3′-TTTGATGTCACGCACGATTTCC-5′

## Abbreviations

COX-2	cycloxygenase-2
ERK	extracellular signal-regulated kinase
IL-6	interleukin-6
IL-1β	interleukin-1β
iNOS	inducible nitric oxide synthase
JNK	c-Jun N-terminal kinase
LPS	lipopolysaccharide
MAPK	mitogen-activated protein kinase
PGE_2_	prostaglandin E_2_
TNF-α	tumor necrosis factor-α
